# Supplementary material for: Phytop: a tool for visualizing and recognizing signals of incomplete lineage sorting and hybridization using species trees output from ASTRAL
Source: Hortic Res. 2024 Nov 21;12(3):uhae330. doi: 10.1093/hr/uhae330 (PMC11879507; doi:10.1093/hr/uhae330)
Supplement: Web_Material_uhae330 [file web_material_uhae330.zip › supp-phytop.pdf]

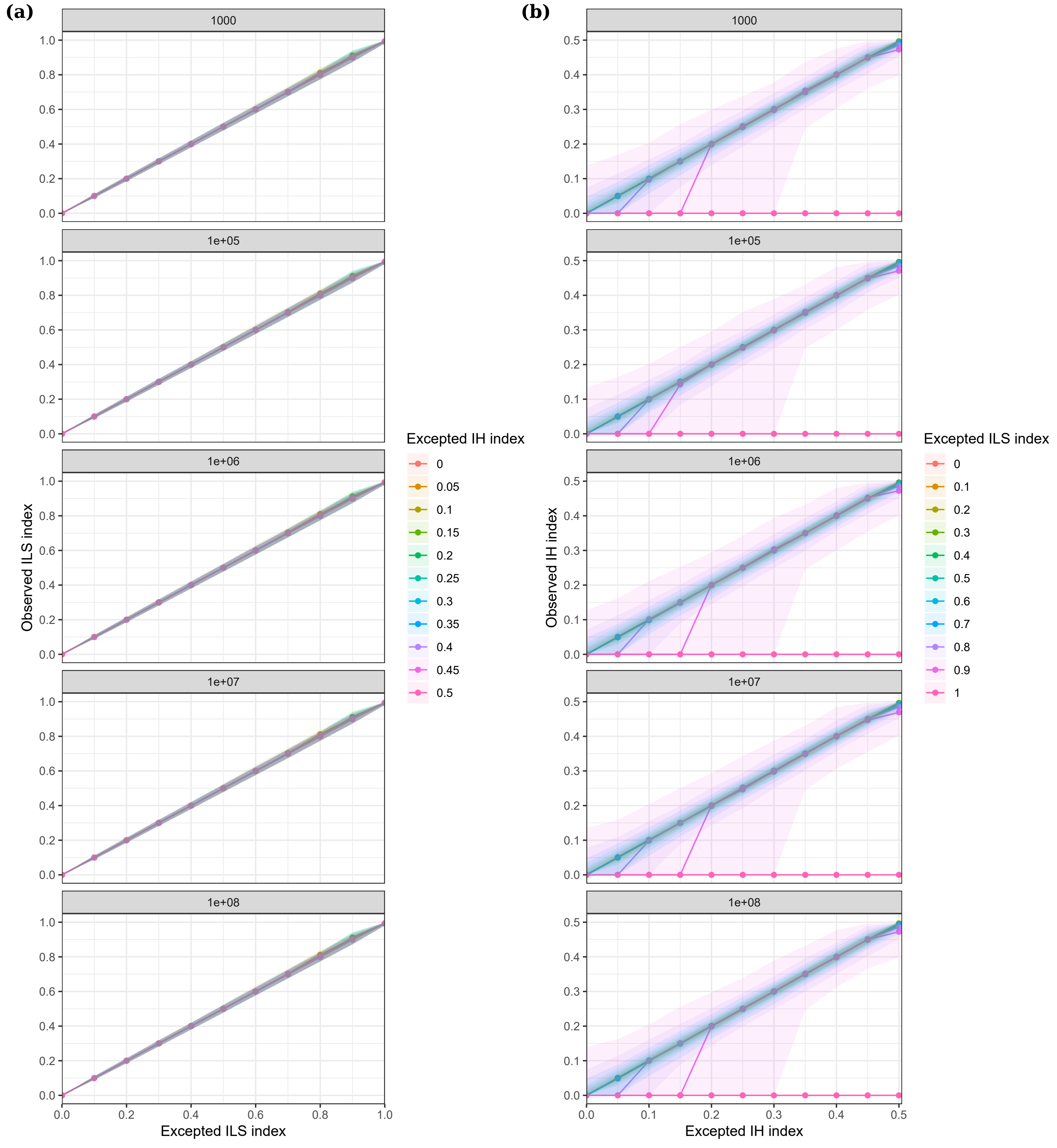

Figure S1. Evaluation of the IH and ILS indices in simulated data using a simple hybridization model with different species divergence times (the number in each gray box). (**a-b**) The distribution of the observed ILS index (**a**) and the observed IH index (**b**) in all simulated data. The shallows indicate 95 % CI of the observed ILS or IH index.

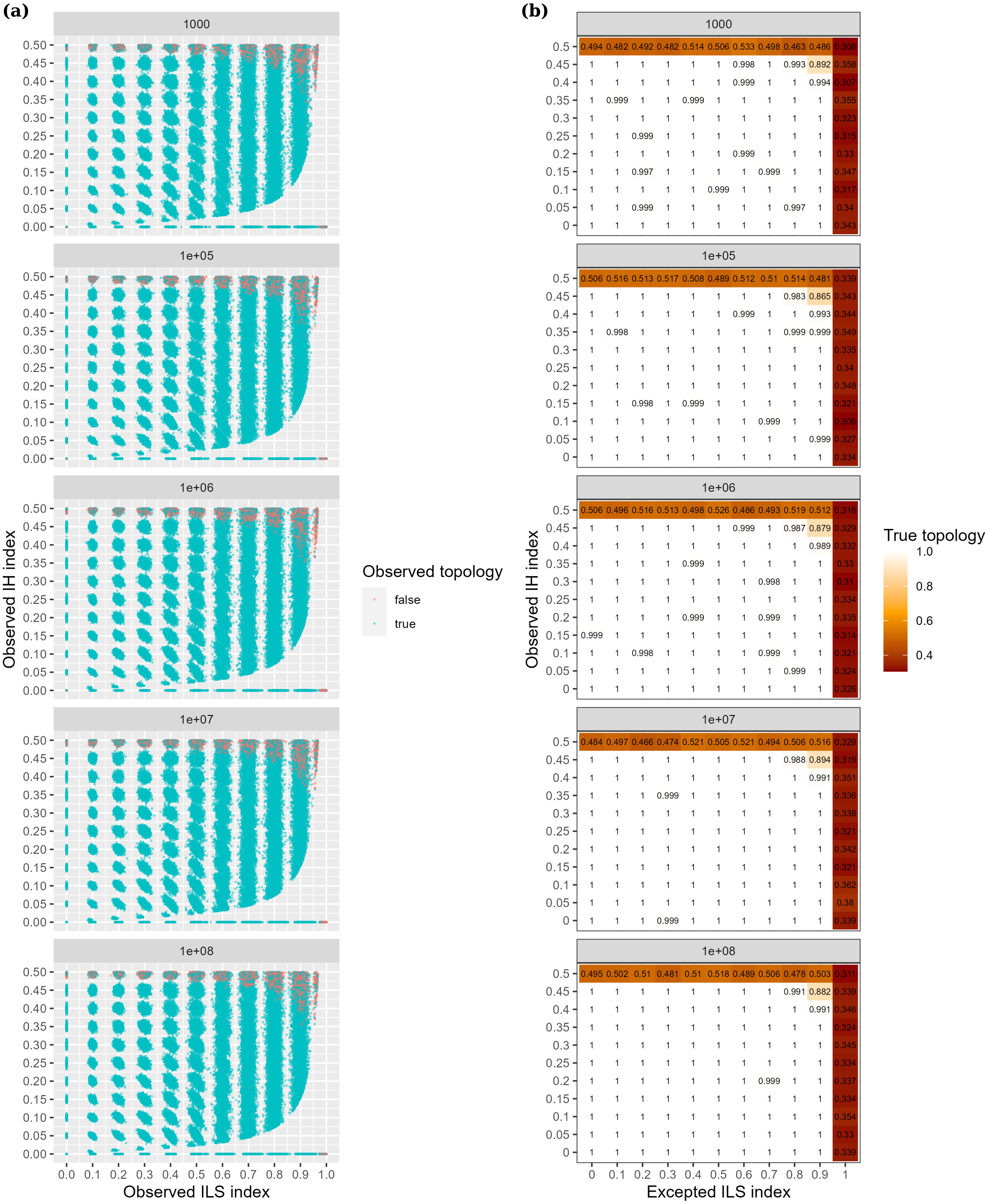

Figure S2. Evaluation of the IH and ILS indices in simulated data under the simple hybridization model with different species divergence times (the number in each gray box). (**a**) The distribution of the observed ILS index and the observed IH index in all simulated data. Red dots represent unexpected topologies, while green dots represent expected topologies. (**b**) The proportions of observed true topologies under different settings of the ILS index and IH index in simulated data.

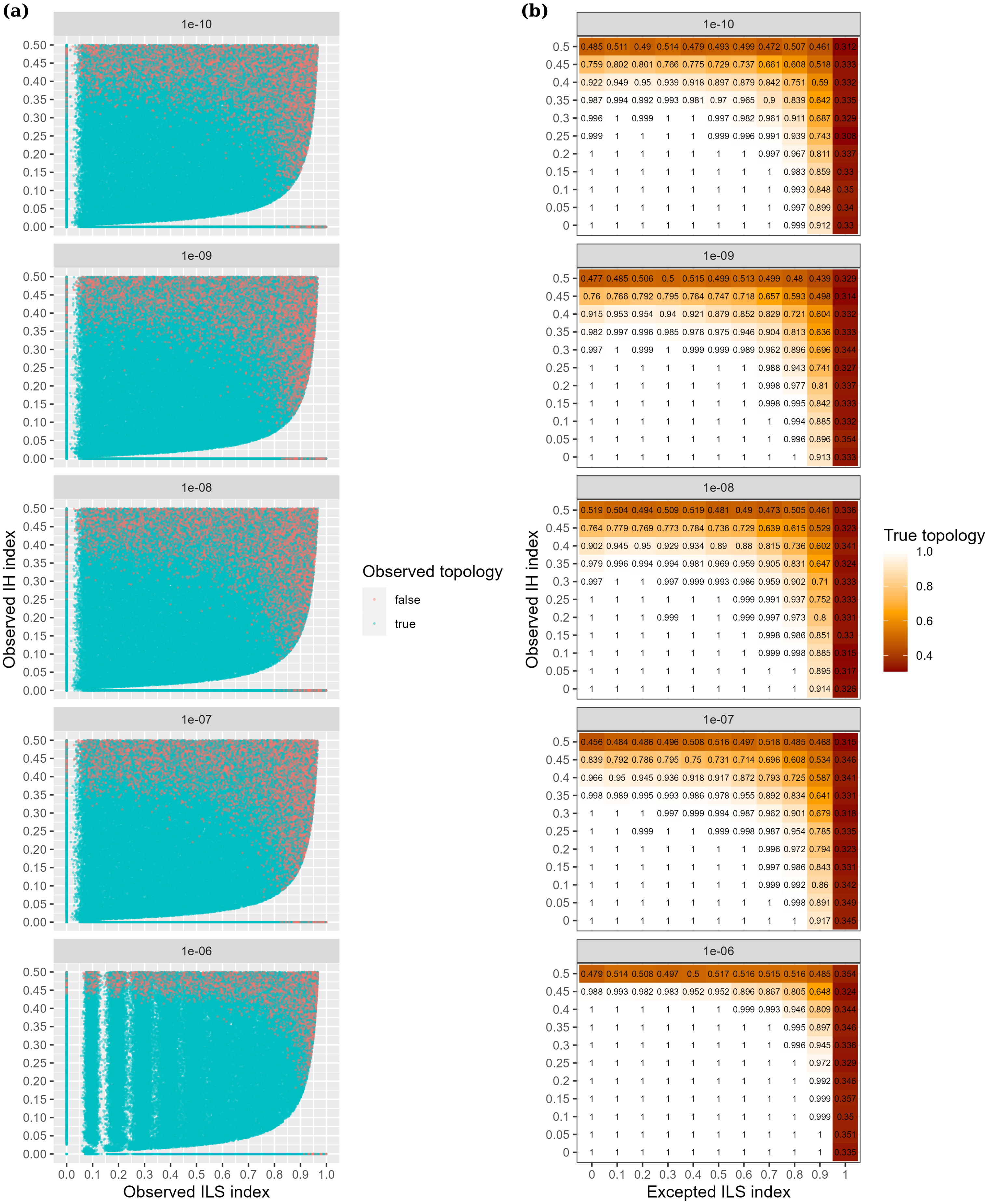

Figure S3. Evaluation of the IH and ILS indices in simulated data under the simple hybridization model with different recombination rates (the number in each gray box). (**a**) The distribution of the observed ILS index and the observed IH index in all simulated data. Red dots represent unexpected topologies, while green dots represent expected topologies. (**b**) The proportions of observed true topologies under different settings of the ILS index and IH index in simulated data.
